# Microstructure Characterization and Corrosion Resistance of Zinc Coating Obtained on High-Strength Grade 10.9 Bolts Using a New Thermal Diffusion Process

**DOI:** 10.3390/ma12091400

**Published:** 2019-04-29

**Authors:** Henryk Kania, Jacek Sipa

**Affiliations:** 1 Silesian University of Technology, Institute of Materials Engineering, Krasińskiego 8, 40-019 Katowice, Poland; 2 Remix S.A., Poznańska 36, 66-200 Świebodzin, Poland

**Keywords:** thermal diffusion coatings, grade 10.9 bolts, corrosion resistance

## Abstract

The article presents the results of research on the application of innovative thermal diffusion zinc coating technology with the recirculation of the reactive atmosphere to high-strength grade 10.9 bolts. The innovation of this method consists in the introduction of reactive atmosphere recirculation and the application of coating powder mix which contains zinc powder and activator. Recirculation of the reactive atmosphere ensures its uniform composition, while the presence of an activator intensifies the process of saturating steel surface with zinc, which boosts the efficiency of active agents. Coatings were created at 440 °C and a heat soaking time of 30–240 min. Coating structure (SEM) was exposed, chemical composition in microsites (EDS) was defined, and coating phase structure (XRD) was identified. The kinetics of coating growth were defined. It was found that the increment of coating thickness was controlled by square root of soaking time. Coatings obtained using innovative thermal diffusion zinc coating technology had a two-layer structure. At the substrate, a compact layer of phase Γ_1_ (Fe_11_Zn_40_) was created, which was covered with a layer of phase δ_1_ (FeZn_10_). The new method of thermal diffusion zinc coating will alow for the creation of coatings of very good corrosion resistance while maintaining strength properties of bolts defined as strength class 10.9.

## 1. Introduction

Due to their versatility and reliability, bolted joints are among most frequent methods of joining steel structures. They demonstrate a number of advantages, which include easy and quick assembly at minimum cost of maintenance and control but also good strength at variable loads [1]. However, due to specific geometry of such joints, they are the most critical area of construction, which in numerous instances necessitates the application of high-strength bolts made of special, additionally heat-treated steel grades.

The durability of steel structures is determined by the impact of the environment whose aggressiveness speeds up corrosive wear. Therefore, adequate anti-corrosion protection of steel structures is an important factor and is decisive for safe exploitation. Steel structures are most often protected against corrosion by hot dip metallization. Zinc coatings are also used to protect bolts designed for joining structural elements [2]. However, the requirement of a proper thread match between the bolt and nut brings about the limitation of coating thickness [3]. Despite being stronger than construction steel, bolts become the weakest element of a structure in a corrosive environment.

Currently, zinc coatings on bolts are obtained by various methods. Hot dip galvanizing (HDG) is most frequently used to protect bolt surfaces [2]. Bolts are galvanized by a batch hot dip method in baskets with centrifugation [4]. Coating consists of a transient layer of intermetallic phase Fe-Zn, which is covered with an outer layer of zinc extracted from the bath [5,6]. The depth of the external layer is difficult to control, while removing an excess of zinc is not always successful at a conventional hot dip galvanizing temperature of 450 °C. Coatings obtained usually have a thickness of 45 to 65 µm. This makes it difficult to obtain a proper fit between bolts and nuts, while decreased coating thickness lowers the strength of the joint compared to other elements of the structure [7]. Much better results are achieved by high-temperature galvanizing. Galvanizing at a higher temperature allows for the effective removal of excess zinc from the surface of bolts. Coating obtained at a high temperature consists exclusively of intermetallic phase Fe-Zn. The thickness of intermetallic phase layers is more effectively controlled with process parameters. The corrosion resistance of high temperature coatings is approximately two times greater than that of conventional zinc coatings [8]. Removal of excess zinc allows one to maintain the thread fit, while increased corrosion resistance yields bolt strength comparable to other structural elements. High temperature galvanizing of bolts is effected at a temperature of ca. 560 °C [9]. In the case of high-strength quenched and tempered bolts, galvanizing at such a temperature poses a risk of compromising the high-strength properties. Furthermore, the pickling procedure in hydrochloric acid, normally applied in the procedure of surface finishing, creates a risk of hydrogen embrittlement due to hydriding in the case of high-strength bolts.

Another method of protecting bolts against corrosion is electro-galvanizing, which ensures obtaining a uniformly thick coating on a threaded surface. Due to small coating thickness (usually up to 15 µm), the corrosion resistance of the bolts, which is significant at the beginning of exploitation, decreases fairly quickly, which considerably shortens the life of the entire structure. The application of galvanized coatings does not require heating of treated elements during the process, which minimizes the risk of high-strength bolts losing their strength after quenching and tempering. However, this method leads to extensive contamination of the natural environment as well as possible hydrogen embrittlement in high-strength steel [10].

Much better results are achieved when zinc coatings on bolts are obtained by sherardizing. The sherardizing process is run in closed rotary retorts in which bolts are placed together with zinc powder and zinc oxide [11]. The retort is soaked at a temperature of 380–450 °C, which allows for the creation of a layer of intermetallic phase Fe-Zn on the bolts [12,13,14]. The protective layer created by sherardizing, similar to the one obtained through high-temperature galvanizing, consists exclusively of intermetallic phase Fe-Zn, which permits the control of case depth and ensures high corrosion resistance. Meanwhile, due to a much lower processing temperature, the high-strength bolts retain their strength properties [13]. It is usually enough to have the surfaces cleaned mechanically before sherardizing in order to prevent hydrogen embrittlement. The major drawback of sheradizing is its relatively long processing time. In order to obtain coatings of the required depth of 15–30 µm, 6 to 12 h will be needed, to be further extended if the process temperature is lowered.

## 2. Innovative Thermal Diffusion Zinc Coating Technology with Reactive Atmosphere Recirculation

The technology of thermal diffusion zinc coating with recirculation of reactive atmosphere is a new technology in zinc coating. The process is run in a closed retort, which is loaded with products to be coated together with powder mixture. The rotating motion of the retort ensures continuous contact of powder mix with the surfaces of processed products. The powder mix is composed of zinc powder, ZnO as a filler, and NH_4_Cl as an activator. In the course of heating the powder mix in the retort, gaseous reaction products are created that move towards the surfaces to be coated. On steel substrate adsorption of zinc atoms created from the gaseous mixture as a result of reduction, dissociation, or exchange occurs.

Forming of thermal diffusion coating results from the chemical reaction of vapor deposition [15,16,17]. Previous studies have shown that NH_4_Cl decomposes according to the following reaction [16]:

*NH*_4_*Cl* → *NH*_3_ + *HCl*, (1)

Zn powder reacts with HCl vapors to form ZnCl_2_ by the reaction:

(2) $$Zn+2HCl\to ZnCl_{2}+H_{2},$$

ZnCl_2_ is reduced by hydrogen to Zn as a result of heating process [16]. During heating, active zinc atoms can also be formed according to Equation [18], as follows:

(3) $$ZnCl_{2}\to Zn+2Cl,$$

The concentration of active Zn atoms in the powder mixture is higher than on the steel surface. The concentration gradient causes internal diffusion of zinc, which in a gaseous form deposits on the steel surface [18].

Active zinc atoms easily diffuse into the steel substrate along existing vacancies, leading to distortion of the crystallographic structure of the steel substrate. The iron atoms on the surface of the substrate can be activated by the following reactions [18]:

(4) $$2HCl+Fe\to FeCl_{2}+H_{2},$$

*FeCl*_2_ → *Fe* + 2*Cl*, (5)

Crystal lattice distortion accelerates the diffusion of active Fe atoms outside [18]. During heating, there is a two-way diffusion between Zn and Fe, causing the formation of Fe-Zn intermetallic phases [17].

The process, besides rotary movement of the retort, is aided by an innovative solution that consists of forced circulation of reactive atmosphere along the retort cylinder [19]. This ensures a better and more uniform contact of product surface with powder mix, both on external and internal surfaces as well as on surfaces of intricate shape. Introduction of atmosphere recirculation along the retort cylinder permits uniformity of reactive atmosphere within the entire volume of the retort, which leads to uniform temperature in the working space, lower consumption of powder mix, and more effective use thereof in comparisation to typical sherardizing [19]. Due to recirculation and better use of active agents, it is possible to create the layers of intermetallic phase Fe-Zn [20] on low-carbon steel at a much shorter time compared to conventional sherardizing technology. At the same time, the coatings obtained through this method demonstrate higher corrosion resistance than the hot dip coatings [21]. The new technology constitutes an alternative to traditional process of sherardizing, in which an activator is not used and mixture motion in the atmosphere is determined solely by the rotating movement of the retort.

## 3. Materials and Methods

Coatings for research were created on a prototype plant equipped with a furnace with a rotary working chamber of capacity 100 kg and forced recirculation of reactive atmosphere. The workload consisted of M10 bolts and threaded bars of strength grade 10.9 made of quenched and tempered 1.7225 steel (42CrMo4). The powder mix was composed of zinc powder with an addition of 15% of zinc oxide ZnO as a filler and 3% of NH_4_Cl as an activator. All that was soaked at the temperature of 440 °C and times of 30, 60, 120, and 240 min. Before filling the working chamber of the furnace, the ingredients of powder mix were dried at the temperature of 120 °C for 12 h in order to reduce moisture content to approx. 1%. The activator was added to the mixture right before processing due to its high hygroscopicity. Before processing, bolt surfaces were cleaned mechanically using sandblasting process to prevent the risk of hydrogen embrittlement.

In order to establish the structure of the thus-produced coatings, metallographic tests were performed with a light microscope. The microstructure and chemical composition in micro-areas of coatings were analyzed with a Hitachi S-3400 N (Tokyo, Japan) scanning microscope equipped with a X-ray energy dispersion spectroscope.

Phase analysis by X-ray diffraction was performed with a JEOL JDX-7S X-ray (Tokyo, Japan) diffractometer using a copper anode lamp (λCuKα = 1.54178 Ǻ) powered with 20 mA current at 40 kV, and with a graphite monochromator. Recording was performed with a stepwise approach of 0.05° step and counting time of 3 s in the range of 10 to 90° 2θ. Phases were identified with the help of the ICDD (Newtown Square, PA, USA) PDF-4+ database. Diffractometric tests were performed on skew-ground sample surfaces so that phase structure could be displayed in the entire cross-section of the coating.

Testing of resistance to the impact of neutral salt spray was performed in accordance with the standard EN ISO 9227 in a salt chamber CORROTHERM Model 610 by Erichsen.(Hemer, Germany) Testing was conducted in a 5% spray of sodium chloride in distilled water at the temperature of 35 °C. In order to determine mass changes, gravimetric analyses were performed in the course of testing following 48, 96, 164, 240, 480, 720, and 1000 h of the exposure of samples in the chamber. Corrosion tests were carried out on grade 10.9 screws with thermal diffusion zinc coating obtained at 4408C and heating time of 12 min.

Tensile strength testing was performed on a tensile strength testing machine Inspekt Table 100 by Hegewald und Peschke MPT GmbH (Nossen, Germany) with maximal load 100 kN.

## 4. Results and Discussion

### 4.1. Surface and Cross-Sections Appearance

**Figure 1:**
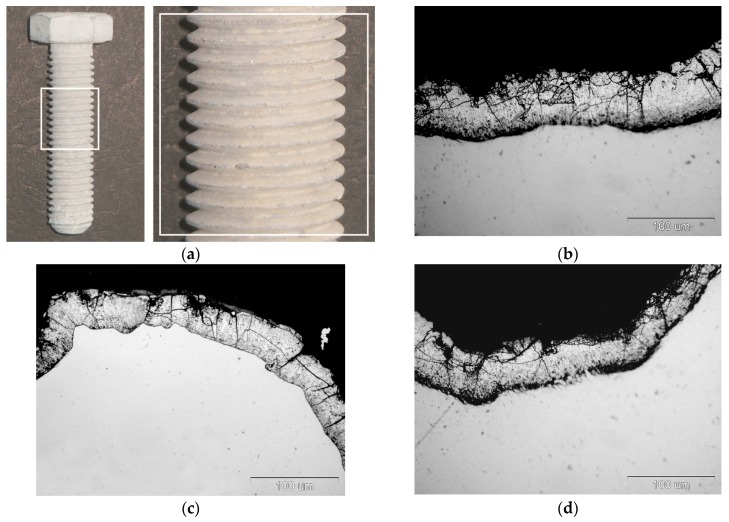
Appearance of surface (**a**) and cross-section of coating on bolt head (**b**), thread tip (**c**), and thread groove (**d**) of grade 10.9 bolt.

External appearance of zinc coatings obtained on a grade 10.9 bolt with the new technology of thermal diffusion zinc coating with reaction of reactive atmosphere is presented in Figure 1. The coatings obtained do not show any discontinuity, while their surface looks matt grey. No traces of powder mix were found on the thread surface after zinc coating (Figure 1a). Additionally, in a cross-section the coating shows no discontinuity and densely covers the bolt surface both on the head (Figure 1b) and on the tip of thread (Figure 1c) as well as in the groove (Figure 1d). The grey and matt appearance of the coating and its cross-sectional morphology are a proof of intermetallic Fe-Zn phases in its structure. The appearance of cracks located mainly in the upper part of the coating can be observed in the coating structure, which may indicate the brittleness of the coating. The occurrence of transverse cracks is characteristic of the intermetallic phases of the Fe-Zn phases.

### 4.2. Growth Kinetics

**Figure 2:**
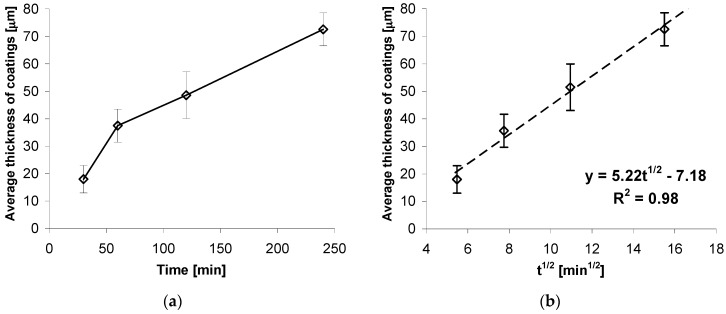
Growth kinetics (**a**) and plot of coating thickness as a function of t^1/2^ (**b**).

Growth kinetics of coatings obtained on high strength grade 10.9 bolts at the temperature of 440 °C and soaking time from 30 to 240 min is presented in Figure 2. Coating thickness increases with the increase of soaking time. However, the increment of coating thickness becomes slower and slower. After 120 min, a coating of average thickness of 50.61 µm was obtained. During further soaking, the increment was over 2 x slower with thickness of 72.62 µm obtained after the time of 240 min (Figure 2a).

The impact of temperature soaking time on coating thickness is shown in more detail as the dependence of thickness in the square root function of soaking time t^1/2^. For the experimentally defined dependence of coating thickness from soaking time, there is a nearly linear correlation of coating thickness against square root of soaking time t^1/2^. The determined trend function and high value of correlation factor R^2^ = 0.98 confirm the linear nature of coating thickness increment. Thus, the growth kinetics for a coating may be described by the following equation:

y (thickness) = 5.22 × t^1/2^ − 7.18, (4)

The presence of a factor equaling t^1/2^ in the equation describing growth kinetics means that the coating increase is a process controlled by diffusion. Since those coatings are created as a result of zinc diffusion into the substrate, it may be concluded that the kinetics of that phenomenon are controlled by square root of time, which appears to be in compliance with literature data [22]. The equation also shows that the growth of the coating begins after the time that is necessary to reach the temperature allowing the diffusion of ingredients.

### 4.3. Microstructure (SEM) and Microanalysis (EDS)

**Figure 3:**
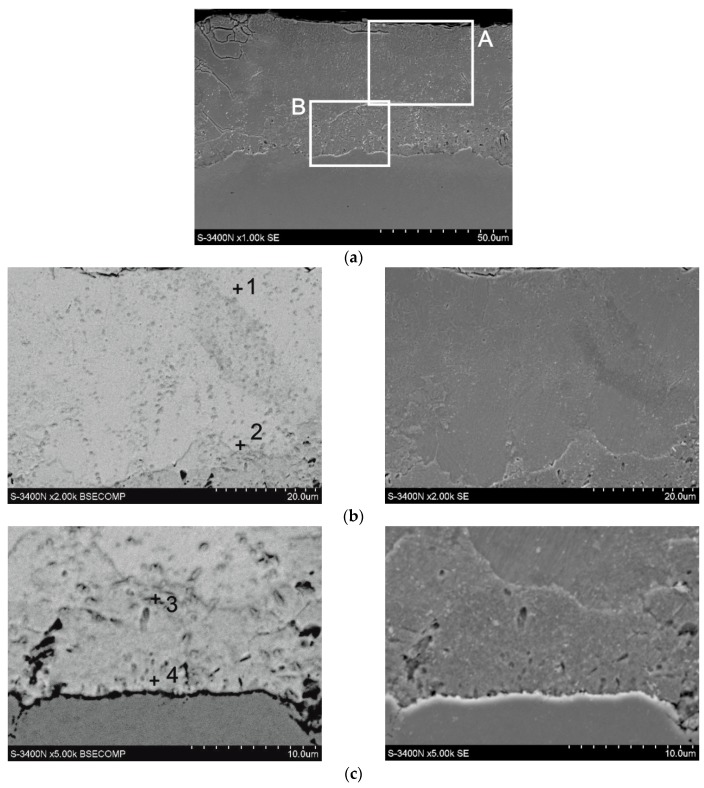
Microstructure (SEM) of coating obtained on high-strength grade 10.9 bolts via thermal diffusion method with reactive atmosphere recirculation: cross-section of the coating with selected areas (**a**), microstructure of the coating in area A (**b**), microstructure of the coating in area B (**c**).

The microstructure of a coating obtained at a temperature of 440 °C and time of 120 min is presented in Figure 3. Percentage contents of analysed elements are shown in Table 1. The coating is composed of two layers: outer layer defined as site A and a layer adjoining the substrate marked as site B. In the outer layer (Figure 3b), the presence of 7.2 wt % Fe and 92.8 wt % Zn was found in outer zone (point 1, Table 1) and 11.4 wt % Fe and 88.6 wt % Zn in the zone bordering on the underlying layer (point 2, Table 1). Chemical composition of outer layer is comprised within intermetallic phase δ_1_ (FeZn_10_) [23]. Change of concentration of components on the cross-section of that phase is typical of phase δ_1_ and may be an evidence of its varied morphology: phases δ_1p_ of palisade structure in the upper zone and phase δ_1k_ of compact structure in the substrate zone [5]. Chemical composition of the underlying layer (Figure 3c) is more homogenous. In the outer layer, there is 19.7 wt % Fe and 80.3 wt % Zn (point 3, Table 1), whereas the substrate layer contains 21.2 wt % Fe and 78.8 wt % Zn (point 1, Table 1). Chemical composition in the underlying layer corresponds to stability range for phase Γ_1_ (Fe_11_Zn_40_) [6].

### 4.4. X-ray Phase Analysis (XRD)

**Figure 4:**
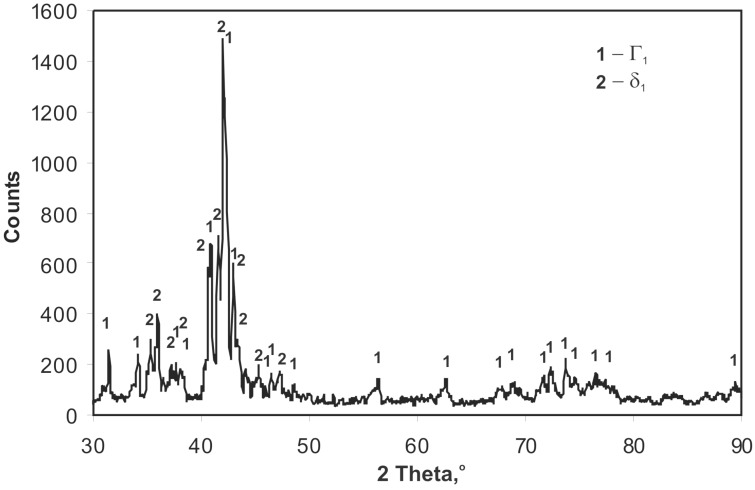
Diffractogram of the surface of skew-ground coating obtained on high-strength grade 10.9 bolts through thermal diffusion method with reactive atmosphere recirculation.

XRD analysis performed on the surface of a skew-ground coating (Figure 4) demonstrates phase δ_1_ (FeZn_10_) and phase Γ_1_ (Fe_11_Zn_40_). Considering chemical composition in the coating microsites (Table 1), it may be claimed that the outer layer of the coating is phase δ_1_, whereas the layer adjacent to the substrate is phase Γ_1_. The research did not confirm any presence of other phase Fe-Zn, although these are stabile in the conditions the coating is created. According to phase equilibrium system, phases Γ (Fe_3_Zn_10_) and ζ (FeZn_13_) are also stabile [24]. Configuration of all stabile phases (Γ, Γ_1_, and δ_1_ i ζ) occurs in the diffusion layer of coatings obtained by HDG method at the temperature of 450 °C [6]. However, the mechanism of producing a hot dip galvanizing coating is more complex than that, and the transition layer is created as a result of simultaneous processes of diffusive growth, solutioning in liquid zinc, and recrystallization [24].

For technologies using powder mixtures as zinc carrier, the available literature does not allow an unambiguous determination of coating phase structure. In conventional sherardizing, a coating is created that is composed of a compact layer of phase δ_1_ (marked as FeZn_7_) and a non-homogenous outer layer of phase ζ (FeZn_13_) [14]. However, Konstantinov [25] claims that the coating is built of phases Γ and δ_1._ Liu [26] obtained a similar structure, making his coatings with a pack cementation method in a powder mix with an addition of activator on 1.7225 (42CrMo4) steel. On the other hand, Wortelen [27] claims that the coating obtained through sherardizing in powder mix with an activator is composed of phases Γ, Γ_1_, δ_1_, and ζ. It must be pointed out that characteristic spectra for phases Γ and ζ coincide largely with the spectra of phases Γ_1_ and δ_1;_ therefore, XRD examination does not allow for unambiguous exclusion of the presence of phases Γ and ζ. However, no other structural components were determined in the microstructure of the tested coating, whose chemical composition might correspond to the range of homogeneity of phases Γ and ζ. This was also confirmed in the research conducted by Chaliampalias [15,28], who obtained a coating composed of phases Γ_1_ and δ_1_ in the pack cementation process.

### 4.5. Corrosion Resistance

**Figure 5:**
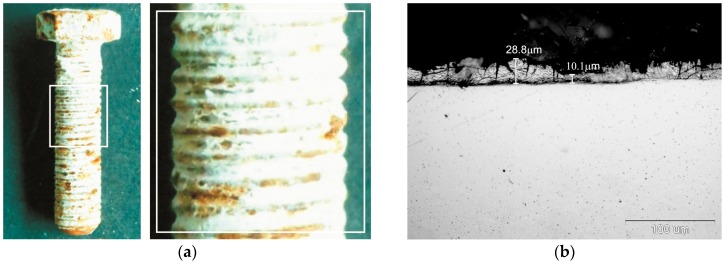
The appearance (**a**) and structure (**b**) of the thermal diffusion zinc coating after corrosion tests in a salt chamber.

The surface appearance of grade 10.9 bolts with thermal diffusion after corrosion testing in neutral salt spray is presented in Figure 5. During exposure in a salt chamber, the thermal diffusion coating becomes covered with products of white rust. After 1000 h of exposure in the salt chamber, local rusty discolouring becomes visible on the surface (Figure 5a). White rust products accumulate in thread grooves and expose the thread tips, where the intensity of corrosion is clearly higher. Upon completion of corrosion test, no distinctive permeation of coating into substrate was visible, which was confirmed by structural research on a cross-section of coating (Figure 5b). After testing, a non-uniform depletion of coating thickness was manifested on the cross-section; however, the coating did not yet lose its continuity. The presence of rusty discolourations on the surface of zinc coatings is characteristic of corrosion of intermetallic phase Fe-Zn. Such a phenomenon is observed during exposure of HDG coatings to an environment that contains chlorides [8].

**Figure 6:**
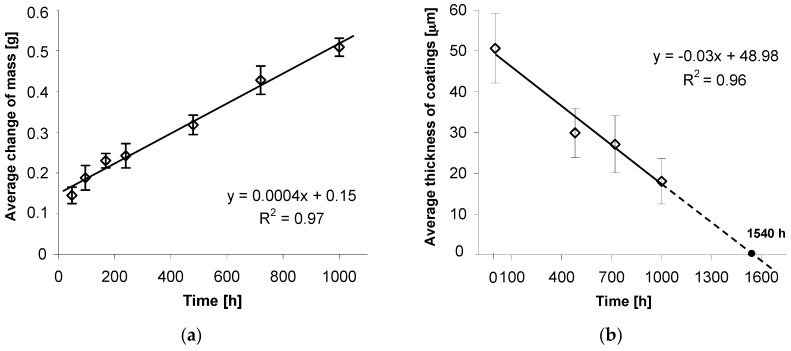
Average change of mass in tested bolts grade 10.9 (**a**) and average change of thermal diffusion coating thickness (**b**) during exposure in salt spray chamber.

Dependence of mass changes in tested coatings from the time of exposure in the salt chamber is presented in Figure 6a. It may be concluded that during exposure to neutral salt spray, the tested coatings are characterized by increment of mass, which is an evidence of accumulation of corrosion products on their surfaces. Changes of bolt mass, and at the same time corrosive wear of thermal diffusion coating, follow a linear relationship. The trend function, determined in the course of experimental research, and the high correlation factor R^2^ = 0.97 (Figure 6a) constitute proper linear match with data and confirm the linear character of the wear of thermal diffusion coating.

The linear character of coating wear is also evidenced by coating loss in its cross-section. Average coating thickness changes after 480, 720, and 1000 h of exposure in a salt chamber are presented in Figure 6b. Assuming average initial thickness of 51.52 ± 8.48 µm, they decreased to 18.03 ± 5.53 µm after a 1000 h corrosion test. The trend function, determined through measurements of average thickness of coating and good match of correlation factor R^2^ = 0.96, confirm the linear character of corrosive wear of coating in its cross-section. Extrapolation of trend line allows an estimation that the total loss of thermal diffusion coating on a bolt’s surface and at the same loss of protective properties will happen after approx. 1540 h of exposure in the salt chamber. In comparison to conventional hot dip galvanizing zinc coatings, the durability of the thermal diffusion coating tested in a corrosion test in a salt chamber [22] results in an approx. 2–3-fold increase in the durability time to penetration of the substrate surface (steel). While carrying out the corrosion test in a salt-spray chamber (acc. EN ISO 9227) traditional hot dip galvanizing coating of comparable thickness (average thickness 50.8 ± 6.42 μm), distinctive permeation of coating into substrate was visible after 480 h of exposure in neutral salt-spray environment [21]. For conventional hot dip zinc coating, much more severe corrosion losses can be observed in the initial stage of corrosion process when the outer layer of zinc is subject to corrosion. When this layer is worn out, the corrosion process proceeds more slowly in the Fe-Zn intermetallic layers [8]. Thermal diffusion zinc coating does not show increased corrosion intensity in the initial stage of corrosion process due to the lack of an outer zinc layer. The corrosion process proceeds only in the Fe-Zn intermetallic layers, whose thickness is higher in comparison with conventional hot dip coatings. The average thickness loss of the coating was used to estimate coating durability. Locally, in areas of lower thickness, the coating may be broken through to the base material. Due to the very good protection properties of the coating and the significant share of areas with a higher coating thickness, the protection of the steel in the initial stages of coating penetration will still be preserved.

### 4.6. Tensile Test

**Figure 7:**
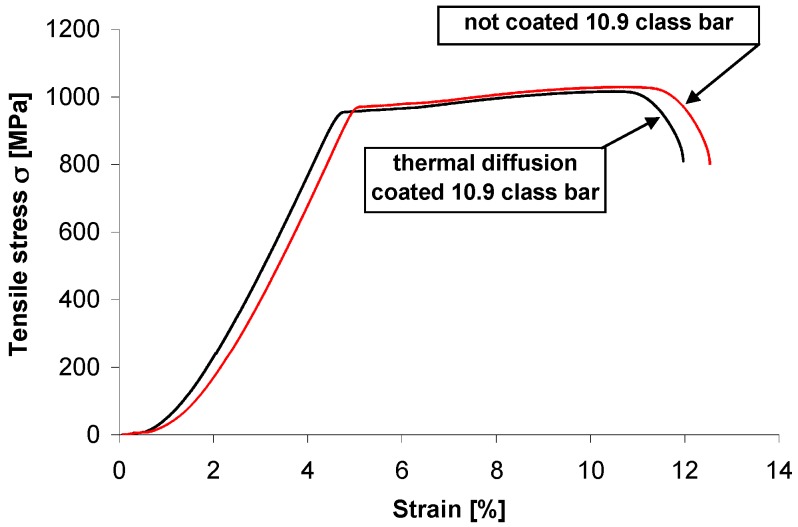
Tensile stress-displacement relationship curve of coated and uncoated 10.9 grade threaded bar.

A diagram of a static tension test on threaded bar grade 10.9 without coating and with thermal diffusion coating is presented in Figure 7. The values of tensile strength limit Rm and conventional yield limit Rp0.2 for three tests on each bar are presented in Table 2. The tensile strength of grade 10.9 threaded bar decreased slightly after creation of thermal diffusion coating. The average tensile strength limit was Rm = 1024 MPa for the uncoated bar and Rm = 1007 MPa for the thermal diffusion coated one, respectively. The process of creating a coating by the new method of thermal diffusion zinc coating did not compromise the tensile strength below the required limit. However, the ratio of conventional yield limit to tensile strength had a similar value of 0.94–0.95 both for the uncoated bar and the thermal diffusion coated one. Tensile strength of the threaded bar coated with the new method of thermal diffusion zinc coating with recirculation of reactive atmosphere fulfilled the tensile strength requirement for grade 10.9.

In these tests, threaded bars M10 in strength grade 10.9 made of 42CrMo4 steel (1.7225) after quenching and tempering process were used. The total length of the sample was 220 mm, gauge length 160 mm. Special high nuts were screwed on the ends of the bar, and then the sample was placed in the holders of the testing machine and stretched until it was broken.

## 5. Conclusions

The innovative technology of thermal diffusion zinc coating with recirculation of reactive atmosphere provides effective anti-corrosion protection for high-strength grade 10.9 bolts while retaining their strength properties. The application of reactive atmosphere recirculation ensured homogenization of its composition and a better contact of product surface with powder mix, which permitted one to obtain continuous coatings of a compact structure in a shorter time compared to the conventional sherardizing method.

The research conducted allows for the following conclusions to be made:

1. The new method of thermal diffusion zinc coating with recirculation of reactive atmosphere permits one to obtain coatings 50–72 µm thick in soaking time of 120–240 min at a temperature of 440 °C upon grade 10.9 bolts;
2. The coatings obtained on grade 10.9 bolts feature a two-layer structure. Directly on the substrate, a compact layer of intermetallic phase Γ_1_ (Fe_11_Zn_40_) is created, which is covered with a layer of intermetallic phase δ_1_ (FeZn_10_);
3. The coatings obtained in this way feature very good corrosion resistance. It is estimated that corrosion strength of 50 µm thermal diffusion coatings on grade 10.9 bolts in neutral salt spray amounts to approx. 1500 h;
4. Thermal diffusion zinc coating parameters permit retaining the strength properties for bolts defined by strength grade 10.9.

## Figures and Tables

**Table 1:** Chemical composition at selected micro-areas of coating obtained on high-strength grade 10.9 bolts (measurement points acc. to Figure 3).

Measurement Point	Content of Elements			
	Fe-K		Zn-K	
	wt %	at. %	wt %	at. %
point 1	7.2%	8.3%	92.8%	91.7%
point 2	11.4%	13.1%	88.6%	86.9%
point 3	19.7%	22.3%	80.3%	77.7%
point 4	21.2%	24.0%	78.8%	76.0%

**Table 2:** Results of tensile strength test of coated and uncoated 10.9 grade threaded bar.

Type of Threaded Bar	Sample Number	R_m_ [MPa]	R_p0,2_ [MPa]	R_p0,2_/R_m_ [MPa]
not coated 10.9 grade bar	test 1	1031.73	980.87	0.95
	test 2	1016.93	955.24	0.94
	test 3	1023.36	968.32	0.95
thermal diffusion coated 10.9 grade bar	test 1	1004.01	946.00	0.94
	test 2	1001.11	936.56	0.94
	test 3	1018.41	962.87	0.95
